# Bioremediation of Per- and Poly-Fluoroalkyl Substances (PFAS) by *Synechocystis* sp. PCC 6803: A Chassis for a Synthetic Biology Approach

**DOI:** 10.3390/life11121300

**Published:** 2021-11-26

**Authors:** Francesca Marchetto, Marco Roverso, Davide Righetti, Sara Bogialli, Francesco Filippini, Elisabetta Bergantino, Eleonora Sforza

**Affiliations:** 1 Department of Industrial Engineering DII, University of Padova, 35131 Padova, Italy; marchetto.francesca95@gmail.com (F.M.); davide.righetti.1@studenti.unipd.it (D.R.); 2 Department of Chemical Sciences, University of Padova, 35131 Padova, Italy; marco.roverso@unipd.it (M.R.); sara.bogialli@unipd.it (S.B.); 3 Department of Biology, University of Padova, 35131 Padova, Italy; francesco.filippini@unipd.it (F.F.); elisabetta.bergantino@unipd.it (E.B.)

**Keywords:** PFAS, PFOS, PFOA, cyanobacteria, bioremediation

## Abstract

One of the main concerns in industrialized countries is represented by per- and poly-fluoroalkyl substances (PFAS), persistent contaminants hardly to be dealt with by conventional wastewater treatment processes. Phyco-remediation was proposed as a green alternative method to treat wastewater. *Synechocystis* sp. PCC6803 is a unicellular photosynthetic organism candidate for bioremediation approaches based on synthetic biology, as it is able to survive in a wide range of polluted waters. In this work, we assessed the possibility of applying *Synechocystis* in PFAS-enriched waters, which was never reported in the previous literature. Respirometry was applied to evaluate short-term toxicity of perfluorooctanoic acid (PFOA) and perfluorooctane sulfonate (PFOS), which did not affect growth up to 0.5 and 4 mg L^−1^, respectively. Continuous and batch systems were used to assess the long-term effects, and no toxicity was highlighted for both compounds at quite high concentration (1 mg L^−1^). A partial removal was observed for PFOS and PFOA, (88% and 37%, with removal rates of about 0.15 and 0.36 mg L^−1^ d^−1^, respectively). Measurements in fractionated biomass suggested a role for *Synechocystis* in the sequestration of PFAS: PFOS is mainly internalized in the cell, while PFOA is somehow transformed by still unknown pathways. A preliminary bioinformatic search gave hints on transporters and enzymes possibly involved in such sequestration/transformation processes, opening the route to metabolic engineering in the perspective application of this cyanobacterium as a new phyco-remediation tool, based on synthetic biology.

## 1. Introduction

Per- and poly-fluoroalkyl substances (PFAS) are man-made organic compounds sharing a hydrophilic head group and a hydrophobic alkyl chain of variable length (from 4 to 16 C) partially (poly-fluorinated) or completely fluorinated (per-fluorinated) [1]. PFAS can be classified according to their carbon chain length (Cn); those with 7 (C7 or C6 in the case of sulfonate ones) or more perfluorinated carbons are referred to as “long-chain” and shorter ones are referred to as “short-chain” [2].

A number of useful characteristics, such as amphiphilic nature, ability to reduce liquids surface tension, high thermal stability, low chemical reactivity, absence of smell and color, high availability, and low cost [3,4], make PFAS ideal for several industrial applications and products, such as firefighting foams, non-stick materials for cookware, or stain high-temperature lubricants, among others [1,5]. All these chemical properties also make them non-biodegradable, widespread, and persistent in the environment, subject to bioaccumulation and biomagnification in the environment [3].

As a fraction <5% is lost in the atmosphere [5], PFAS contaminate the soil, the aquatic environment, and drinking water at concentrations ranging from pg L^−1^ to µg L^−1^, and up to g L^−1^ in areas close to industrial sites [6]. The high degree of PFAS biomagnification along the food chain caused the contamination of the environment and the inhabiting fauna, generating chronic direct and indirect exposure. In Veneto, a north-eastern region of Italy, PFAS are accounted for a contamination case-study, since they present at high concentrations both in surface and groundwater (up to µg L^−1^) and in drinking water, with consequent human health impairment due to the significant exposure of the population living in this region [7,8].

Perfluorooctanoic acid (PFOA) and perfluorooctane sulfonate (PFOS), are the most known C8 congeners of the two main sub-classes of PFAS, i.e., carboxylic and sulphonic acids. PFOA and PFOS result to have the criteria for persistence, biomagnification and long duration of transnational transport to be included in the definition of Persistent Organic Pollutants (POPs), according to the Stockholm Convention [5], and PFOA is classified as Substances of Very High Concern [9]. This compound is also toxic for reproduction (Cat. 1B), has carcinogenic potential (Cat. 2 of the European Chemicals Risk Assessment Committee Agency), and it has a residence time of 3.8 years in human blood and breastmilk [10]. These compounds are well absorbed orally, poorly eliminated and distributed in a tissue-dependent manner, due to their high binding affinity to serum albumin and fatty acid-binding proteins, mainly in serum, kidney and liver [11,12]. In humans, detectable levels of PFOS and PFOA have been reported in blood [8], in the umbilical cord, and in many organs [13,14], where levels may vary due to various factors [3].

Several epidemiological studies proved the correlation between PFAS exposure and health effects because these compounds can cause countless different diseases affecting all important human organs and compartments [15]. They can cause immunotoxicity altering immune and thyroid function [16], hepatotoxicity with multiple liver disease, and neurotoxicity [16,17]. Moreover, the exposure leads to defects in embryonic development and infant mortality with damages to the reproduction system [14,16] and to other adverse effects as lipid and insulin dysregulation and cancer [15].

PFAS removal from the environment relies on different techniques: adsorption by granular activated carbon filters [18] and ion exchange resins are used for the temporary remediation actions to pollution [1,16], as well as membranes for filtration or separation, such as Reverse Osmosis and Nanofiltration. Incineration and sonolysis were also proposed to destroy or transform these compounds [1,16]. Lab-scale experiments about electrochemical, sonochemical, advanced oxidation processes, and plasma are potentially effective approaches for both long-chain and some short-chain PFAS [19].

Cheap, energetically competitive, and effective techniques for PFAS removal from the wastewater are not available yet, as available techniques are associated with exceedingly high energy, capital, and operational costs [19].

New approaches should be proposed accordingly, where groundbreaking multidisciplinary solutions might be complementary with the current efforts to limit the environmental impact of PFAS pollution. Bioremediation seems to be an environmentally friendly approach to PFAS removal, requiring less harsh conditions than other remediation techniques [20], and potentially reducing operational costs [21], and it is suitable for both polluted water and soil. Even though some organisms, such as bacteria, fungi, and plants, showed to be able to remove PFAS, it is still not clear the possibility of applying bioremediation for such compounds [22]. Synthetic biology also offers a new methodology/contribution to deal with emerging pollutants [23]. Bioreactors, providing a confined and controlled environment, are the best choice when dealing with genetically engineered microorganisms [24], since in situ bioaugmentation strategies suffer from severe ecological and regulatory concerns [25].

Among organisms suitable for bioremediation purposes, cyanobacteria combine two relevant features, as they: (i) can survive in a wide range of habitats and (ii) represent convenient and attractive platforms for carbon-neutral production processes [26]. These photosynthetic prokaryotes exploit CO_2_ relying only on water, light, and some micronutrients and can be applied also for remediation of industrial pollutants [27]. This is particularly interesting in the case of wastewater poor in biodegradable COD (chemical oxygen demand), as carbon can be easily obtained from CO_2_. As it may occur in conventional wastewater treatment plants, the C:N ratio, is not a constraint. Among other species, model cyanobacterium *Synechocystis* PCC 6803 (*Synechocystis* hereafter) is a promising candidate, with a fully sequenced genome allowing to perform comparative analyses and to follow up plastic adaptations and genome variation by NGS resequencing. The availability of the complete genome (inferred proteome) sequences makes this organism suitable for prediction-driven design and selection via in silico screens.

In this work, *Synechocystis* was evaluated for its possible use in PFAS phyco-remediation, and, to our knowledge, this is the first report of the cultivation of such a species for PFAS bioremediation. The growth capabilities of *Synechocystis* were assessed in the presence of PFOA or PFOS at different concentrations in a controlled system under both short-term and long-term exposure. Even though this species was already cultivated in wastewater media, confirming its resistance to several effluent streams [28], in this work, a controlled environment with synthetic water was applied, to avoid possible interference by variable surrounding media and environmental conditions. Respirometric tests were carried out to assess the survival capabilities of *Synechocystis* to PFAS contamination in the case of short-term exposure. Long-term effects at high concentrations of these compounds were then evaluated in continuous and batch reactors, and the removal capabilities in *Synechocystis* ascertained. A preliminary search for putative enzymes was also carried out to assess potential degradation pathways and identify possible metabolites.

## 2. Materials and Methods

### 2.1. Organism, Growth Medium, and Equipment

The strain used for all the experiments is *Synechocystis* sp. PCC 6803 from the Pasteur Culture Collection (PCC, Paris, France) of Cyanobacteria. BG11 [29] was used as growth medium and modified in some cases as reported in Section 2.3. The species was maintained in Petri dishes with 1% Agar BG11, while liquid preinocula were cultivated in 100 mL Erlenmeyer flasks at 30 °C and at 50 μmol photons m^−2^ s^−1^ of light intensity, provided by white LED lamps. Preinocula were then used for both batch and continuous experiments.

PFOA and PFOS potassium salt (Sigma Aldrich, St. Louis, MO, USA, purity 96% and >98%, respectively) were added at 1 mg L^−1^ concentration. This high concentration was chosen based on the process proposed in Section 3.

In batch and continuous experiments, light was provided at 150 μmol photons m^−2^ s^−1^, by a CF GROW LED lamp, model CXB3590-X1 of CREE LED with 380–780 nm spectrum. Light intensity was measured by a Delta OHM photoradiometer (Delta Ohm, Padova, Italy, HD 2102.1).

Daily pH measurement was performed using a HI 9124 pH meter (Hanna instruments, Woonsocket, RI USA), on a 3 mL sample. Magnetic stirring and continuous bubbling of air with 5% CO_2_ (*v*/*v*) were applied to ensure mixing and non-limiting carbon supply in both batch and continuous experiments. pH control was made thanks to the buffer capacity of the CO_2_-bicarbonate system.

Batch and continuous experiments were carried out to understand the possible toxic effect of PFAS on algal growth.

Batch experiments were carried out in Quickfit ^®^ Drechsel bottles of 250 mL (working volume 170 mL, Sigma-Aldrich, St. Louis, MO, USA) and 5 cm diameter with insufflation spout. The experiments started from OD_750_ = 0.4 and were carried out in duplicates at a constant temperature of 30 °C, under continuous light (150 μmol photons m^−2^ s^−1^).

Continuous experiments were carried out in polycarbonate reactors of volume $V_{R}$ = 350 mL (5 cm of thickness), with an inlet for nutrients: fresh medium was continuously provided through a Sci-Q 400 peristaltic pump (Watson Marlow, Clinton, MA, USA) at a constant volumetric flow rate, Q (mL d^−1^), from a sterilized, external, stirred glass bottle. An overflow tube was placed on the opposite side of the fresh medium inlet, ensuring the same volumetric flow rate, thus keeping constant the culture volume. It is possible to approximate the system to a continuously stirred tank reactor (CSTR), as proven by tracer experiments (not shown). An internal septum in the reactor prevents a short cut-off flow of incoming nutrients and outlet biomass. Reactors were kept at a constant temperature of 30 °C, under continuous light of about 150 µmol photons m^−2^ s^−1^. After a transient period, the continuous system reached a steady state. Cell concentration, dry weight and nutrients consumption were measured, as described in Section 2.2, for at least 5–6 days after the steady state was reached. The reactor was operated continuously at set hydraulic residence time (*HRT*) of 2.44 days, calculated as (Equation (1)):

(1) $$HRT=\frac{V_{R}}{Q}.$$

At steady state, volumetric biomass productivity can be calculated as the ratio between the outlet biomass concentration $C_{x}$ on the residence time (Equation (2)):

(2) $$P_{x}=\frac{C_{x}}{HRT}.$$

The areal biomass productivity can be calculated as (Equation (3)):

(3) $$P_{A}=\frac{C_{x}}{HRT}\cdot W,$$

where *W* is the reactor depth in meters.

### 2.2. Respirometric Tests

The respirometric protocol proposed in this work is an adaptation of a previous protocol [30], which also describes the respirometric apparatus, and it was applied to ascertain possible short term toxic effects.

To ensure non-limiting nutrients conditions, in each respirometric test, the biomass inoculum was resuspended in fresh BG11 medium. The medium pH was buffered at 7.5 with sodium carbonate (at a concentration corresponding to 0.2 mg L^−1^ of carbon), and the value was checked during the test. To avoid carbon limitation and lower the dissolved oxygen concentration to about 6 mg L^−1^, pure CO_2_ was bubbled in the device before each respirometric run. The duration of the light: dark cycles was chosen as sufficient to obtain enough Dissolved Oxygen (DO) measurements (one acquisition every 15 s) to retrieve the values of Oxygen Production and Consumption Rates (OPR and OCR, respectively). Once measured the OCR and OPR in a PFAS-free medium, each compound was separately added at increasing concentrations up to 1 mg L^−1^ of PFOA and PFOS, and the effects of oxygen variation were registered. For each compound, the tests were carried out in double/triple biological replicate, by repeating the entire protocol starting from different inocula.

A second (batch cultivation) and third (continuous cultivation) series of experiments was carried out to assess the effect of higher concentrations of PFOA and PFOS (1 mg L^−1^), in BG11, added separately.

### 2.3. Analytical Measurements

Cyanobacterial growth was measured daily as optical density at 750 nm, by double-beam spectrophotometer UV-Visible UV 500 (Spectronic Unicam, Mamhilad, UK) in both batches and continuous experiments.

Dry weight (DW) was measured gravimetrically on biomass filtered with 0.2 µm nitrocellulose filters (Whatman^®^, Sigma-Aldrich, St. Louis, MO, USA), previously placed for 15 min at 105 °C to eliminate humidity. The filter with the sample was then placed at 100 °C for 2 h and finally weighed (Atilon Acculab Sartorius Group^®^, Göttingen, Germany). DW was measured at the beginning and the end of batch experiments, while, in CSTR, samples were measured daily at steady-state.

Nitrate (NO_3_) and ammonia (NH_4_) and orthophosphate concentrations were determined on filtered samples using Hydrocheck Spectratest kits by Reasol^®^ (Milano, Italy) to verify the possible nutrient limitation.

PFAS were measured by high-resolution mass spectrometry in flow injection analysis mode (FIA-HRMS) considering inlet or initial water samples ($C_{i,e\;}$), where the subscript *i* refers to the component, and *e* to the initial or inlet concentration, and the liquid fraction of filtered culture samples ($C_{i,ext}$) at the end of batch experiments, and in the outlet, at steady-state, in a continuous system.

To preliminarily understand the destination of PFAS compound in *Synechocystis* biomass, the culture sampled at the end of batch experiments and in the outlet of the steady-state continuous system were completely disrupted, and PFAS were measured in the mixture $\left(C_{i,lys}\right)$.

%*R_i_* is the fraction of compound removed from the medium and not found any longer in the supernatant, calculated as (Equation (4)):

(4) $$\%R_{i}=100*\frac{C_{i,e}-C_{i,ext}}{C_{i,e}}.$$

The fraction $\%C_{i,bio}$ is calculated as (Equation (5)):

(5) $$\%C_{i,bio}=100*\frac{C_{i,lys}-C_{i,ext}}{C_{i,e}}$$

and corresponds to the fraction of PFAS which was internalized by the biomass but released after cell disruption.

The fraction which is not detectable anymore in the sample $\left(\%D_{i}\right)$, i.e., neither found in the supernatant nor in the lysate, can be calculated as (Equation (6)):

(6) $$\%D_{i}=100*\frac{C_{i,e}-C_{i,lys}}{C_{i,e}}\;.$$

The removal rate $r_{i}$ and the areal removal rate $r_{Ai}$ were based on the $\%R_{i}$, calculated as (Equations (7) and (8)):

(7) $$r_{i}=\frac{C_{i,e}-C_{i,ext}}{HRT},$$

(8) $$r_{Ai}=r_{i}*W.$$

Samples were homogenized by a SHM1 Homogenizer (Stuart Equipment, Stone, UK), centrifuged at 12,000× *g* for 10 min, and diluted 1:10 with acetonitrile spiked with the internal standard (perfluorononanoic acid, 1 mg L^−1^). The FIA-HRMS system was equipped with an Ultimate 3000 UHPLC chromatograph coupled with a qExactive hybrid quadrupole-Orbitrap mass spectrometer (Thermo Fisher Scientific, Waltham, MA, USA). FIA was carried out at 0.1 mL min^−1^ flow rate, using water/acetonitrile 1:1 as eluent. The MS conditions were set as follows: electrospray (ESI) ionization in negative mode, resolution 35,000, AGC target 1 × 10^6^; max injection time 200 ms, scan range 100–500 Da. The capillary voltage was 2.8 kV, capillary temperature was 280 °C, and auxiliary gas was nitrogen at 40 a.u. Calibration was performed with a standard solution from Thermo Fisher Scientific (Pierce™ ESI Negative Ion Calibration Solution; Waltham, MA, USA). MS data were analyzed with Xcalibur 4.0 software (Thermo Fisher Scientific, Waltham, MA, USA).

**Figure 1:**
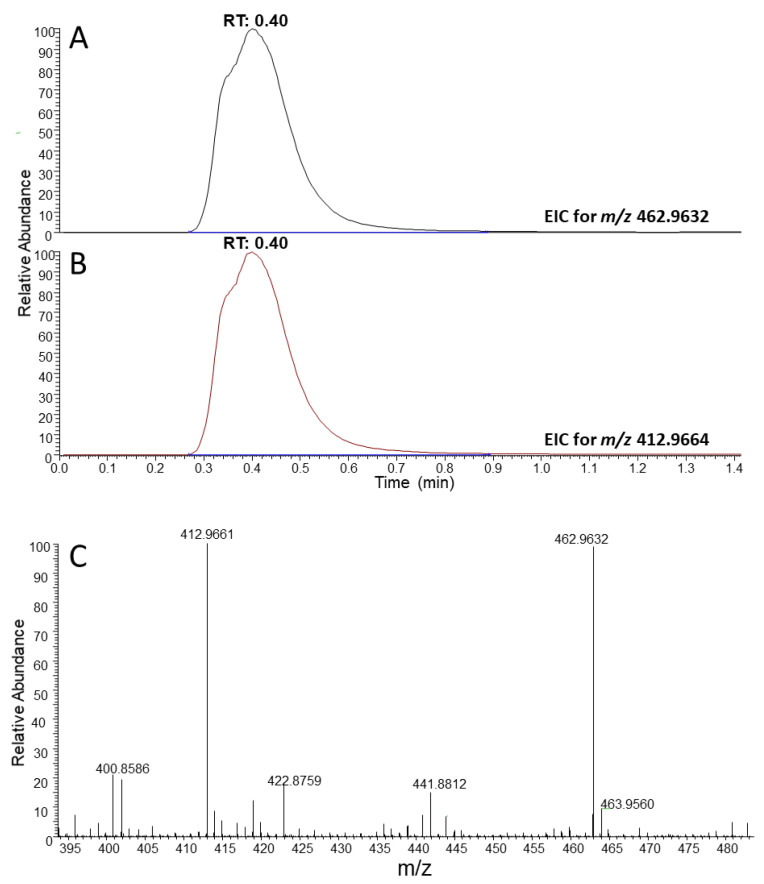
Representative chromatogram obtained from the analysis of a sample with 1 mg L^−1^ of PFOA by FIA-HRMS. (**A**) Extracted ion chromatogram (EIC) for the internal standard (perfluorononanoic acid, *m/z* 462.9632). (**B**) EIC for PFOA (*m/z* 412.9664). (**C**) Mass spectrum of PFOA and PFNA. Mass accuracy < 5 ppm.

Analytes were determined by the integration of the pseudo-chromatographic peak related to the EIC chromatogram (extracted ion chromatogram) obtained by selecting the exact mass relative to the deprotonated ion [M-H]^-^ of the analyte of interest, with a mass accuracy < 5 ppm (Figure 1). Detection limit was 1 µg L^−1^ for both PFOA and PFOS. Blank samples were always processed in the same batch of analysis, as cross contaminations of PFAS are a known unwelcomed drawback. The precision of the method, evaluated from the reproducibility of the IS area between analysis, was good and showed relative standard deviations (RSD%) < 20%. The combined effect of method recovery and matrix interference, evaluated by comparing the area of the IS in samples and in water solutions spiked at the same concentration, was higher than 90%.

To measure the PFAS internalized in the biomass, cell lysis was carried out by disruption using Beadbeater: aliquots of cell culture were mixed with Glass beads (150–212 mm, Sigma G1145-100G, Sigma-Aldrich, St. Louis, MO, USA) in 2 mL vials and 3 cycles of 30 s were applied, with ice incubation between each cycle.

The possible presence of PFAS bond to the cell debris was further ascertained: lysates from the previous method were centrifuged (12,000× *g*, 10 min), and pellets were treated with 1 mL of a methanol:chloroform 2:1 solution, sonicated for 10 min, and centrifuged (12,000× *g*, 10 min). The supernatant was dried under nitrogen flow and reconstituted with 1 mL of acetonitrile spiked with IS and analyzed by FIA-HRMS.

The suspect analysis aimed at identifying potential PFOA and PFOS metabolites was performed by UHPLC coupled to HRMS. Chromatographic separation was achieved by a Kinetex EVO C18 column 100 mm × 2.1 mm, 2.6 µm (Phenomenex, Castel Maggiore, Italy) at flow rate 0.25 mL/min and temperature 20 °C. The elution was performed in a gradient mode (0–6 min 0% B, 6–15 min 0% B to 100% B, 15–20 min 100% B, equilibration time 10 min), using water as eluent A and acetonitrile as eluent B, both 5 mM NH_4_OH. Some MS parameters were accordingly modified: AGC target 3 × 10^6^, scan range 50–750 Da. The capillary voltage was 2.9 kV, the capillary temperature was 320 °C, auxiliary gas, and sheath gas was nitrogen at 40 and 20 a.u., respectively. In UHPLC-HRMS analysis the limit of detection was 100 ng L^−1^ for both PFOA and PFOS.

In silico prediction of possible PFOA and PFOS metabolites was performed by Biotransformation Mass Defects software (Agilent Technologies, Palo Alto, CA, USA).

### 2.4. Statistical Analysis

ANOVA test (Analysis of Variance), followed by post-hoc Siegel-Tukey test, was carried out to ascertain statistically significant differences in respirometric tests and for PFAS removal. Non-significantly different data were grouped by the same letters in the graphs. T-student test was applied on biomass concentration as steady-state in the case of PFOA and PFOS addition, compared to the control.

### 2.5. Bioinformatics

The screening of *Synechocystis* spp inferred proteomes was performed using BLASTp with default settings, except for the Organism field, restricted to *Synechocystis*. As query sequences, only proteins with experimentally demonstrated function were used. The reciprocal blast hit approach was used to exclude false orthologues.

## 3. Results and Discussion

PFAS are persistent pollutants for which effective techniques for rapid detection and effective control are still missing [31], despite the ascertained hazards to the environment and human health. Integrated, multidisciplinary research is, therefore, urgently required to develop new approaches for bioremediation, and this work aims at evaluating the potential of *Synechocystis* in remediation of wastewaters with a wide range of PFAS load.

In wastewater treatment, microalgae can mediate degradation of organic contaminants either directly (via native pathways) or indirectly (via recombinantly expressed enzymes). This prompted many laboratories to develop new tools for algal cultivation, “omics” analyses, and synthetic biology [32]. For the design and the construction of an efficient synthetic microbial scavenger, three initial steps were underlined, consisting of (i) the essential choice of the chassis organism, (ii) the verification of its tolerance to the specific toxic compound/s, and (iii) the selection of the enzyme toolbox and/or the metabolic pathway to be eventually engineered [33].

In this framework, the cyanobacterium *Synechocystis* is a model organism [26], able to survive in complex and polluted wastewater [28]. To further confirm its possible usefulness as a scavenger for phyco-remediation, the toxic effects on this microorganism of PFOA and PFOS with short- and long-term experiments were ascertained, based on respirometry and batch and continuous cultivation.

### 3.1. Assessment of Short-Term Toxicity of PFOS and PFOA at Low and High Concentrations on Synechocystis sps PCC6803

Respirometric tests were carried out with *Synechocystis* in the presence of PFOA and PFOS, separately, at increasing concentrations. Similar respirometry techniques are conventionally used in the environmental field to assess toxicity, also in photosynthetic organisms [34]. Two ranges of concentrations were tested: low (up to 46 µg L^−1^) and high (in the order of mg L^−1^), to cover the ones usually found in wastewaters from various countries [35], and be even much larger than those occurred in extremely contaminated waters of the Veneto region (up to 50 µg L^−1^ in industrial wastewaters and to 2–3 µg L^−1^ in superficial water bodies; data from ARPAV report [36]), as a representative example of a recent contamination by PFAS.

These analyses allowed to observe in a short time (3–6 h) the rates of oxygen production (OPR) and consumption (OCR) as a function of the increasing concentrations of each compound. The results of each cycle were compared with a control cycle in the absence of PFAS. No significant OPR/OCR variation with respect to the control (up to 46 µg L^−1^, data not shown) suggests *Synechocystis* is not affected by the presence of such compounds at low concentration.

**Figure 2:**
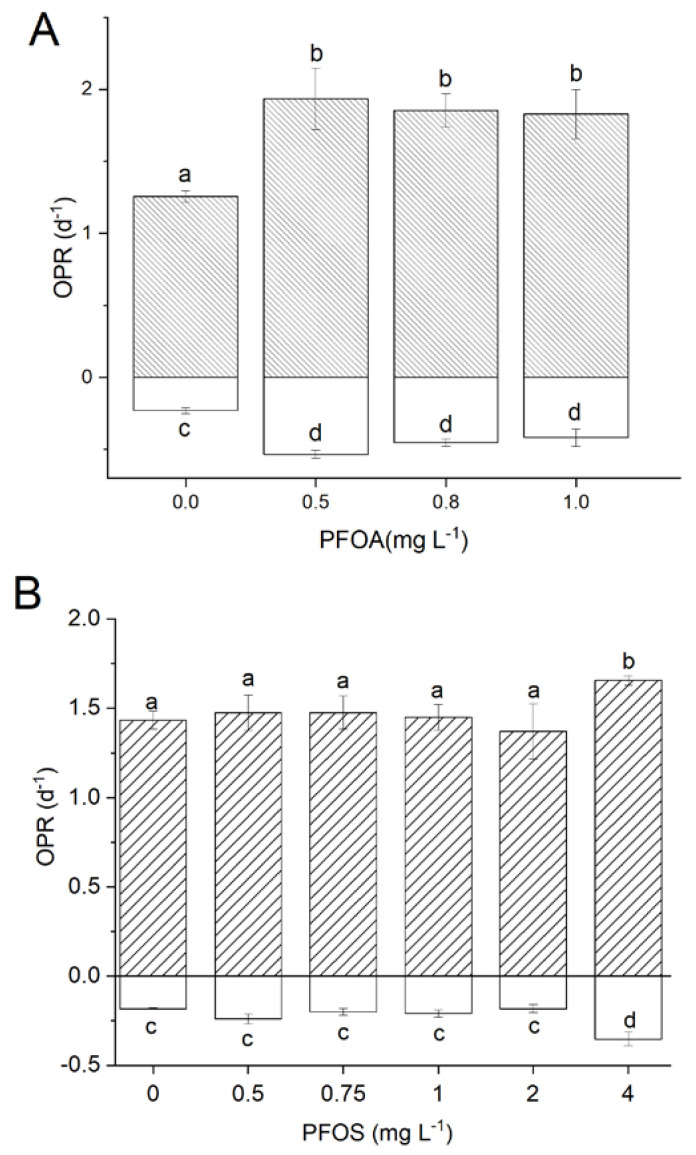
Toxicity test based on respirometry as a function of PFOA (**A**) and PFOS concentration (**B**). Error bars refer to standard deviation and letters indicate statistically significant results for each series: data that share the same letter were grouped according to Tukey’s test. Data that share the same letter are not statistically different.

Other experiments demonstrated the absence of short-time toxic effects, even under high concentrations of each compound (Figure 2). Conversely, increased OPR/OCR values were observed, along with increasing concentration of the compound. A significant increase in both photosynthesis and respiration was obtained at concentrations > 0.5 mg L^−1^ PFOA, while the analysis was extended up to 4 mg L^−1^ PFOS, since no effect was registered with the same concentrations (Figure 2). The increase in respiration is of special interest in axenic microalgal cultures, as it was reported to be related to the mixotrophic metabolism in *Synechocystis* [28].

In summary, basing on respirometric tests [34], concentrations of the compounds up to >0.5 mg L^−1^ for PFOA and up to 4 mg L^−1^ for PFOS did not reveal any short-term toxic effects. On the contrary, an unexpected increase in oxygen evolution was observed, possibly suggesting a change in the metabolic equilibrium between respiration and the photosynthetic processes, a result that certainly deserves further investigations to dissect the mechanisms involved and clarify whether such compounds are actually metabolized and/or up taken by this algal species.

### 3.2. Mid- and Long-Term Effect of PFAS Exposure on Synechocystis Growth

Since neither toxicity nor inhibition effect were observed in short-term, the possible effects of PFOA and PFOS on biomass in both mid- and long-term exposure were investigated. Two sets of experiments were carried out at PFAS concentrations of 1 mg L^−1^, similar to values observed in the undergrounds water nearby the industrial polluted site [37]. Growth rate and contaminant consumption were measured in two different growth conditions.

#### 3.2.1. Effect of PFOA and PFOS in Batch Experiments

**Figure 3:**
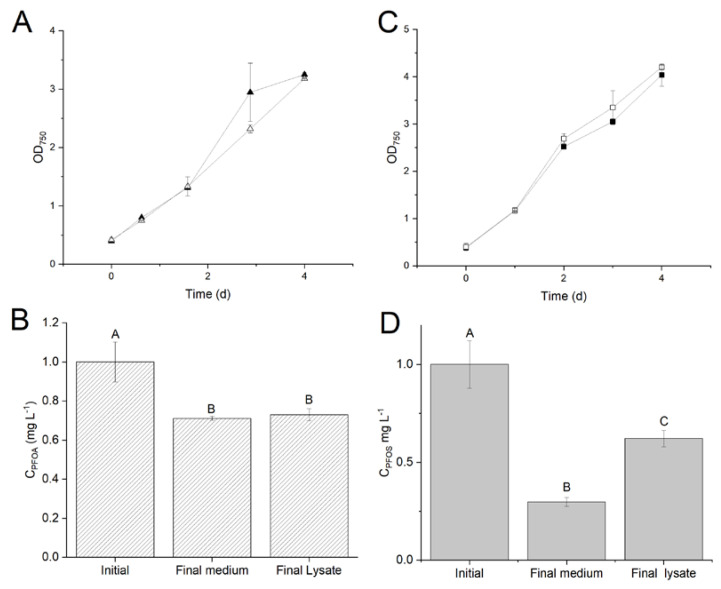
*Synechocystis* growth (**A**,**C**) and PFAS concentration variation (**B**,**D**) in the absence (open triangle/square) or presence (full triangle/square) of 1 mg/L PFOA (**A**,**B**) or PFOS (**C**,**D**). Error bars refer to standard deviation. Letters represent statistically significant differences, according to Tukey’s test: data that share the same letter are not statistically different.

The first set of growth experiments was based on batch curves, to confirm the absence of toxicity by the PFAS supplied. No significant differences between the control and the batches with 1 mg L^−1^ of PFOA or PFOS (Figure 3) in terms of optical density were ascertained. For each compound, a control without the contaminant was carried out simultaneously, to account for the biological variability of the pre-inoculum, a typical drawback of batch experiments.

In samples taken on the first and last day of exposure, removal of PFOA and PFOS from the external medium grew up to 28% and 71%, respectively ($\%R_{i}$, Figure 3B,D). To shed light on such *Synechocystis* mediated removal of these two compounds, biomass samples were homogenized, to release the cytosolic materials and compare the concentration of the compounds (*i*) in the lysate, $C_{i,lys}$, with that measured in the external medium, $C_{i,ext}$. No difference in concentration between $C_{PFOA,lys}\;$and $C_{PFOA,ext}$ was observed (Figure 3B), confirming that PFOA removed from medium (%*D_PFOA_* was 28%) was not present inside the cells. On the other hand, $C_{PFOS,lys}$ was found to be higher than $C_{PFOS,ext}$ (Figure 3D), suggesting that cell lysis provokes the release of a fraction of PFOS ($\%C_{PFOS,bio}=32\%$). *Synechocystis* cells are, thus, capable of removing both PFOA and PFOS from the external medium, either by uptake into the cytosol or entrapment it in the membrane systems [36]; then, PFOS is partially accumulated inside the cell, while PFOA is not found in the lysates, eventually having been transformed.

It should be stressed that batch systems are not the best growth system, as (i) main variables (nutrients, light and biomass concentration) change over time, and (ii) batch growth is strongly influenced by the acclimation of pre-inocula, which, in turn, may affect the removal rate of the compounds by the cells, particularly related to the composition of the membranes and the regulation of possible transporters. Indeed, it was previously shown that batch experiments do not allow a complete acclimation of the cells [38], also affecting the mass balances calculation.

#### 3.2.2. Experiments in a Continuous Reactor

To overcome aforementioned variability, while possibly highlighting limitations due to acclimation phenomena, three photobioreactors were operated under continuous conditions at a residence time of 2.44 days. One reactor was fed by BG11 as a control, while the other media contained, respectively, a constant concentration of PFOA (1 mg L^−1^) and PFOS (1 mg L^−1^). Continuous systems allow overcoming the variability related to the pre-inoculum, as the cells are cultured under stable environmental conditions and continuously fed, ensuring their acclimation [39].

All the reactors easily reached the steady-state after 12–15 days, with constant biomass concentration, confirming that *Synechocystis* is not affected by a long-term presence of PFAS in the medium. Interestingly, an even increased biomass was observed in the presence of PFOS: a concentration of 0.51 ± 0.02 g L^−1^ was obtained at steady-state, with respect to the 0.41 ± 0.02 g L^−1^ of the control and a 0.40 ± 0.03 g L^−1^ in the case of PFOA addition (Table 1).

**Figure 4:**
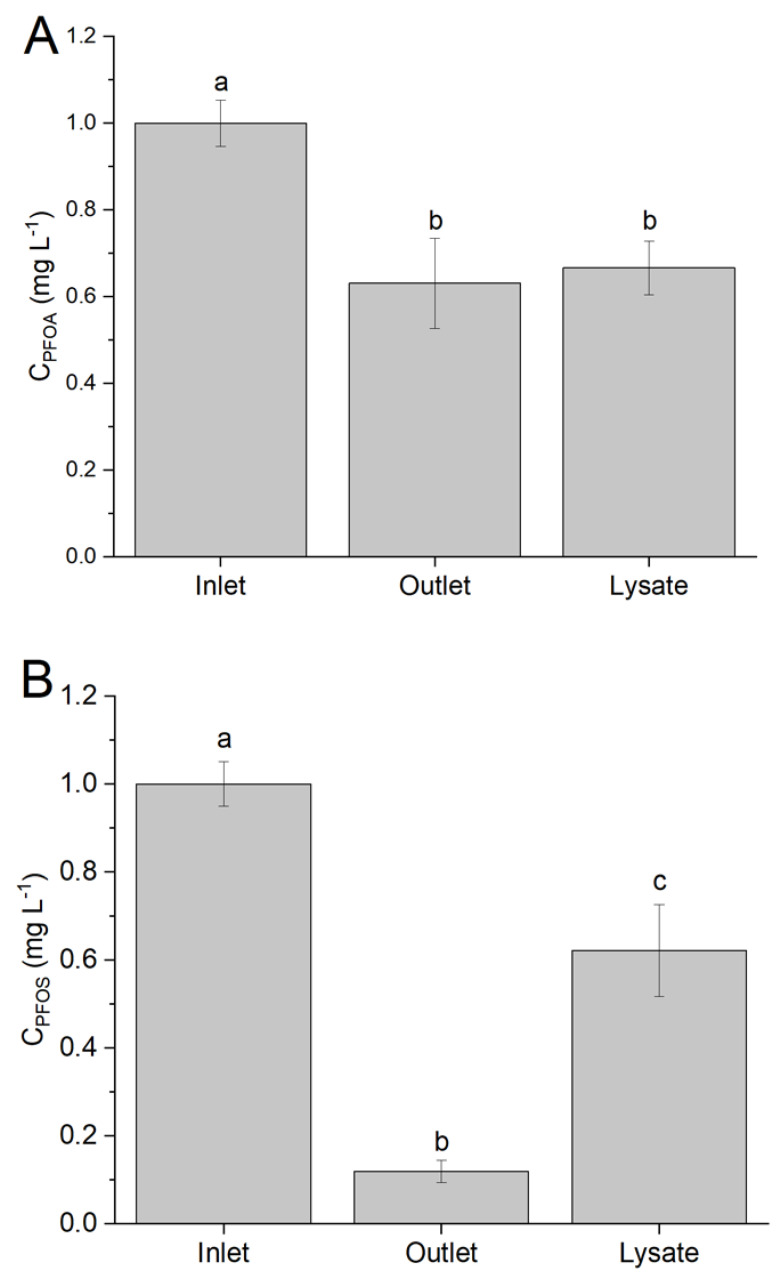
PFAS analysis of samples taken from continuous system fed with 1 mg L^−1^ PFOA (**A**) and PFOS (**B**). Inlet refers to the concentration provided, outlet is the concentration in the external medium in the overflow, and lysate is the concentration of culture disrupted by bead beater. Error bars represent standard deviation of replicates, while letters refer to statistically significant difference. Data sharing the same letter were grouped according to Tukey’s test.

A PFOS and PFOA removal was confirmed in the continuous system, for both $C_{i,lys}\;$and $C_{i,ext}$ (Figure 4). In the case of PFOA (Figure 4A), a 37% reduction ($\%R_{PFOA}$) in final concentration was measured, and only a very small fraction of the compound was detected in the lysate ($\%C_{PFOS,bio}=3.6\%$), confirming that PFOA is effectively removed from the external medium and cannot be found in the cellular cytosol. *Synechocystis* was able to remove around 88% of the PFOS (Figure 4B) present in the medium ($\%R_{PFOS}$), but roughly half of it was then found in the soluble lysate ($\%C_{PFOS,bio}=51\%$). Consequently, a fraction of the PFOS initially present was not detected ($\%D_{PFOS}=49\%$), eventually entrapped by the membranes in the pellet.

To ascertain this possibility, pellet samples were treated by a more exhaustive extraction, using a methanol/chloroform solution (Section 2.3) before proceeding to the analysis. FIA-HRMS measurements showed that PFOS partially adhered to the membrane fraction, while PFOA was not detected in the extracted samples. Due to the matrix effect in the analytical determination of water-soluble PFAS and the fraction attached to membranes, it is not possible to quantitatively compare the amount of compounds recovered by solvent extraction. Nevertheless, this suggests PFOS might interact with the membrane structures, in agreement with other reports on increased membrane permeability in the presence of PFOS [40,41].

In summary, even though *Synechocystis* was effective in the removal of both compounds from the medium, this appeared to occur via different mechanisms: PFOS was partially internalized as present in the cytosol and partially adsorbed by the membrane, while PFOA was possibly actively metabolized. Comparing the results of biosorption (i.e., including both absorption and adsorption processes by biological organisms) with those obtained in the batch system, it is confirmed that acclimation has occurred under continuous cultivation, increasing the removal rate. Accordingly, it appears reasonable to conclude the active role of *Synechocystis* in PFAS removal.

Growth experiments performed both in batch and in continuous reactors in the presence of up to 1 mg L^−1^ of one compound at a time, resulted in higher biomass concentrations than those measured in the respective control reactors, together with the partial removal of PFAS from the external medium.

Limiting our analysis to the latter observation, some preliminary considerations were made: from the mass balance point of view, in batch reactors, a maximum of 28% for PFOA and 71% for PFOS were removed from the external medium, corresponding to a removal rate of about 0.07 and 0.18 mg L^−1^ d^−1^, respectively. In continuous cultivation, higher removal efficiency was observed, up to 37% for PFOA and 88% for PFOS, with a removal rate of 0.15 and 0.36 mg L^−1^ d^−1^. These results highlight the different efficiency in removing PFAS relatively to the cultivation system used: in a continuous system, after setting the operating conditions, a process of acclimation of the organism is firstly observed, and after some time the steady-state is achieved [38]. Continuously grown cells are more acclimated than those grown in the batch system, where cells are subjected to changing conditions, both in terms of medium composition (as substrates are consumed and products released) and of light captured by the photosynthetic complexes (which is reduced as cell density increases).

To our knowledge, the effect of PFAS on *Synechocystis* has not been assessed yet, even though toxicity data are available for other photosynthetic microorganisms as freshwater microalgae, such as *Selenastrum capricornutum* [42], *Chlamydomonas reinhardtii* and *Scenedesmus obliquus* [43], and *Anabaena* [44]. Recent papers focused on understanding the possible role of PFAS in cellular toxicity for microalgae belonging to the *Chlorella genus*: Niu et al., [45] observed a toxic effect on *Chlorella* sp., as well as a reduction of catalases and oxidases. Li et al. [46] also observed downregulation of the genes involved in the photosynthetic metabolism in *Chlorella pyrenoidosa*, as a response to PFOA exposure. This means that even evolutionarily close species can suffer different toxicity effects for the same compound due to species-specific physiological differences. Evidence from this work suggest this cyanobacterium as a good chassis for further adaptation and/or manipulation procedures aimed at producing a good scavenger organism for PFOA and PFAS.

From an engineering perspective, the possibility of using *Synechocystis* for bioremediation is interesting, even though some considerations should be made: as the range of PFAS concentration in wastewater is extremely wide, removing very low concentrations of contaminants may result in not feasible reactor volumes and residence times. A first step aimed at concentrating the compound is reasonable, to reduce the volumes of water to be treated. This is easily obtainable by using adsorption or filtration units [47], followed by a desorption/counter filtration unit, which may allow recovering a more concentrated stream, treatable in a biological reactor [48]. Another approach presented in the literature suggests the possibility of a biological regeneration of the spent adsorbent [48], which is not proved to be applicable for photosynthetic organisms. Preliminary calculations, anyway, suggest that the application of cyanobacteria is promising as, from our results, an extrapolated areal removal rate of about 7 and 18 mg m^−2^ d^−1^ for PFOA and PFOS removal, respectively, were calculated. These data, even if preliminary, are an interesting starting point, when compared to the removal rate of common phytodepuration plants (1.4 g of PFAS per hectare per year [49]). Certainly, this is just a preliminary and potential calculation, laying the fundamentals for future research and feasibility assessment. On the other hand, *Synechocystis* proved to be able to grow and survive in the presence of high concentration of PFAS, posing promising perspectives.

### 3.3. Search for Synechocystis Proteins Possibly Involved in PFASs Transport and Metabolism

To better evaluate the possibility of active transport of PFAS in *Synechosystis*, a bioinformatic approach was applied. At least so far, only animal (human and rat) transporters have been investigated for the capability to carry PFAS, but some clues also exist for bacteria [41,50]. PFOS entrance into eukaryotic cells has been imputed mostly to fatty acids-binding liver proteins, organic anions transporters (OATPs) and Na+/taurocholate co-transporting polypeptides (NTCPs); NTCPs and OSTα/β activity from humans and NTCPs and OATPs activity from *Rattus norvegicus* have been characterized [51]. Aquaporins have been recently suggested as a possible gateway for PFAS in plants [52].

The overall activity of animal, plant or bacterial protein toward PFAS has yet to be clarified. In addition to transporters, some enzymes have to be considered, which are known to degrade PFAS to some extent: e.g., *Armoracia rusticana* peroxidase [53] and *Pleurotus ostreatus* laccase [54] are known to be active as fluoroacetate dehalogenases [55].

A preliminary bioinformatic analysis (see Section 2.5) was performed to highlight the presence of orthologues for these proteins in the *Synechocystis* genus. Proteins noted as bile acid: sodium symporters, ABCs associated proteins, fatty acid CoA ligases and aquaporins are present in various compartments in the *Synechocystis* cell, potentially acting as a gateway for PFOS. Further enzymes, similar to laccases and fluoroacetate dehalogenases, were retrieved, offering a clue about the possible transport and degradation players. Retrieved candidates are summarized in Table 2, a complete version of which (including all accession numbers) is presented in the Supplement to this article.

Since their presence has been assessed mostly at a genus level, and not specifically for *Synechocystis* PCC 6803, further investigation is needed to better determine candidate identities and numbers in the model cellular system used in this work.

### 3.4. Search for Possible PFAS Metabolites

As both PFOA and PFOS partially showed a decrease of concentration from the medium, a survey on possible metabolites was performed by using HRMS features on samples exhibiting the largest removal rates. The high selectivity and sensitivity ensured by the HRMS system allow highlighting signals that can be ascribed to structural modifications of the original PFOA and PFOS molecules, such as decarbonylation, reductive defluorination, and trifluoromethyl loss, as ions as modified species are still ionizable under ESI conditions. Possible modifications were simulated by an in-silico approach by Biotransformation Mass Defects software, which suggests loss or addition of atoms as reported in Table 3. The presence of the related [M-H]^−^ ions was searched for by extracting the accurate mass values with a maximum tolerance of 5 ppm. Results showed that, in the experimental conditions used, no relevant signals were evidenced for the selected modifications. The survey was also extended to shorter-chain PFOA and PFOS congeners, as suggested by Huang et al. [56], but, also, in this case, no signals were detected. It has to point out that the loss of the functional groups, i.e., sulfonic or carboxylic ones for PFOS and PFOA, respectively, does not allow forming species that can be efficiently ionized by the ESI-MS system in the negative ion mode [53,56,57]. Other experiments have to be specifically planned to identify possible degradation products, also with complementary detection systems.

Considerations of the different structures of PFOA and PFOS, together with the data from the chemical analysis, can suggest a rationale for the different fates observed with these compounds in *Synechocystis* cultures.

The amphiphilic properties of PFOS significantly increase the permeability of microalgal membranes [40], and this might be truthfully extrapolated to *Synechocystis*. Membrane transporters identified in the genome-inferred proteome (Table 2) may also have a role in the internalization of the compound into the cell. Since, after cell lysis, the molecule can be detected also in the methanol/chloroform treated insoluble fraction, it is possible to hypothesize a trapping or storage process due to the chemical affinity with the lipid bilayer of the wide membrane system. As for PFOA, instead, lack of detection in any cellular fraction suggests its transformation by one (or more) unknown enzyme(s). No metabolites were identified by the suspect HRMS analysis, implying that modifications that occurred were different from the hypothesized ones. In this context, a deeper untargeted analysis is required, also based on other ionization techniques complementary to ESI, to cover a wider range of potential metabolites with different chemical properties.

The most likely potential candidate enzymes for PFOA biotransformation were found to be laccases and dehalogenases. The former class of enzymes, together with peroxidases, are currently under the magnifying lens of researchers investigating biocatalysis as a tool to relieve recalcitrant pollutants, PFAS included, from contaminated waters [58]. In this field, protein engineering is increasingly exploited to obtain enzymes endowed with high operative stability, as well as a wide substrate assortment. Indeed, putative laccase enzymes, probably involved in decarboxylation and decarbonylation of PFOA and PFOS, were found in the inferred proteome of *Synechocystis* (Table 3). Even though fluoroacetate dehalogenases characterized so far can only mediate defluorination of PFAS having at least one hydrogen atom at the alpha carbon of the molecule [55], the defluorination ability of *Synechocystis* dehalogenases is yet unknown; in addition, these enzymes might be unable to mediate a direct defluorination of PFOA and PFOS, while being able instead to defluorinate intermediate products from laccases activity. The hypothesis that *Synechocystis* cells might be capable by themselves of phyto-remediating wastewater from PFOA and PFOS remains to be verified.

## 4. Conclusions

PFAS are a class of molecules raising increasing concerns from an environmental perspective. In this work, a possible bioremediation approach was proposed. *Synechocystis* was grown in PFAS enriched media, showing, for the first time, its capability of surviving in such conditions and suggesting an active role of this species in the removal of perfluorinated compounds: growth was not inhibited by PFOA and PFOS in a range concentration of mg L^−1^, much higher than those occurring in contaminated waters. A removal up to 37 and 88% for PFOA and PFOS, respectively, was observed, suggesting that this species is able to import the two compounds. Some of the transporters preliminary identified by bioinformatic search might sustain such import, which was never reported in the previous literature. A fraction of the compounds removed from the medium is retained in the cytosol, partly attached to the membrane, but a net portion was not detected in the suspension. Although no relevant signals of predicted metabolites were currently found, the evidenced degradation rate suggests planning other specific experiments. These results open a possible new approach for phyco-remediation of PFAS, showing that a bioremediation approach is promising to reduce the pollution by perfluorinated compounds.

## Figures and Tables

**Table 1:** Summary of data obtained in the continuous system of the control, compared to those fed by PFOA and PFOS. Data with asterisks * are significantly different with respect to the control, according to T-student test.

	Control	PFOA	PFOS
$\mathrm{Biomass}\;\mathrm{concentration}\;(g_{x}L^{-1}$)	0.41 ± 0.02	0.40 ± 0.03	0.51 ± 0.02 *
OD [-]	1.44 ± 0.1	1.36 ± 0.04	1.81 ± 0.06 *
$\mathrm{PFAS}\;\mathrm{specifically}\;\mathrm{removed}\;\left(mg_{i}g_{x}^{-1}\right)$	--	0.925 ± 0.41	1.72 ± 0.32
$\mathrm{Biomass}\;\mathrm{productivity}\;(mg_{x}L^{-1}d^{-1}$)	170 ± 8.3	160 ± 8.9	210 ± 8.23 *
$\mathrm{Areal}\;\mathrm{biomass}\;\mathrm{productivity}\;(g_{x}m^{-2}d^{-1}$)	8.5 ± 0.4	8.0 ± 0.38	10.5 ± 0.41 *
$\mathrm{PFAS}\;\mathrm{removal}\;\mathrm{rate}\;(mg_{i}L^{-1}d^{-1}$)	--	0.15 ± 0.06	0.36 ± 0.07
$\mathrm{Areal}\;\mathrm{PFAS}\;\mathrm{removal}\;\mathrm{rate}\;(mg_{i}m^{-2}d^{-1}$)	--	7.5 ± 3	18.1 ± 3.52

**Table 2:** Candidate *Synechocystis* proteins possibly involved in PFASs transport and/or metabolism. A complete version of this table, reporting all accession numbers, is included in the Supplementary Material file. * Found in *Synechocystis* PCC 6803.

Name	Biochemical Function	Sequence(s)	Similarity (%)
Na+-taurocholate cotransporting polypeptide (NTCP)	Bile acid: sodium symporter	8	27–29% to Human NTCP, NTCP2, NTCP4 and NTCP7.
ATP binding cassette transporter C family member (ABCC)	ATP-binding cassette domain-containing protein	40	23–42% to *Arabidopsis thaliana* ABCC2
	ABC transporter ATP-binding protein	44	23–36% to *A. thaliana* ABCC2
	peptidase domain-containing ABC transporter	5	23–27% to *A. thaliana* ABCC2
	type I secretion system permease/ATPase	2	25–28% to *A. thaliana* ABCC2
	ABC transporter *	3	27–29% to *A. thaliana* ABCC2
	TOBE-like domain-containing protein	2	30–34% to *A. thaliana* ABCC2
	Multidrug resistance ABC transporter ATP-binding and permease protein	1	36% to *A. thaliana* ABCC2
	cyclic nucleotide-regulated ABC bacteriocin/antibiotic exporter protein	1	26% to *A. thaliana* ABCC2
	ATP-binding protein of ABC transporter	1	31% to *A. thaliana* ABCC2
Aquaporin (aqp)	Aquaporin	8	37–100% to *Synechocystis* sp. PCC 6803 aqpZ
Laccase	Multicopper oxidase domain-containing protein	1	24% to *Pleurotus ostreatus* laccase POX1
Fluoroacetate dehalogenase	Alpha/beta hydrolase	19	25–39% to *Burkholderia* sp. Fac-dex
	Alpha/beta fold hydrolase	53	22–40% to *Burkholderia* sp. Fac-dex

**Table 3:** List of possible PFOA and PFOS metabolites predicted by Biotransformation Mass Defects software (Agilent Technologies, Palo Alto, CA, USA), with their relative HRMS signals, and candidate enzyme activities likely to mediate the modification.

Modification	Atom Loss	Result Formula	[M-H]-	Candidate Enzyme
PFOA		C8 H F15 O2	412.9664	
Decarboxylation	-C-O2	C7 H F15	not detectable is ESI condition	Laccase
2× Reductive Defluorination	+H2-F2	C8 H3 F13 O2	376.9853	Fluoroacetate dehalogenase
Trifluoromethyl Loss	-C-F3+H	C7 H2 F12 O2	344.979	
Decarbonylation	-C-O	C7 H F15 O	384.9715	Laccase
Reductive Defluorination	+H-F	C8 H2 F14 O2	394.9758	Fluoroacetate dehalogenase
Oxidative Defluorination	-F+H+O	C8 H2 F14 O3	410.9708	Fluoroacetate dehalogenase
PFOS		C8 H F17 O3 S	498.9302	
2× Reductive Defluorination	+H2-F2	C8 H3 F15 O3 S	462.9491	Fluoroacetate dehalogenase
Decarbonylation	-C-O	C7 H F17 O2 S	470.9353	Laccase
Trifluoromethyl Loss	-C-F3+H	C7 H2 F14 O3 S	430.9428	Laccase
Reductive Defluorination	+H-F	C8 H2 F16 O3 S	480.9396	Fluoroacetate dehalogenase
Oxidative Defluorination	-F+H+O	C8 H2 F16 O4 S	496.9346	Fluoroacetate dehalogenase

## Supplementary Materials

The following are available online at https://www.mdpi.com/article/10.3390/life11121300/s1, Table S1. Candidate *Synechocystis* proteins possibly involved in PFASs transport and/or metabolism. * Found in *Synechocystis* sp. PCC 6803.

## Nomenclature

*V_R_*	Volume of the reactor [mL]
*C_i,e_*	Concentration of compound found in inlet or initial water samples [mg L^−1^]
*C_i,ext_*	Concentration of compound found in the filtrated sample at the end of batch experiment or in the outlet [mg L^−1^]
*C_i,lys_*	Concentration of compound found in the lysate sample at the end of batch experiment or in the outlet [mg L^−1^]
*C_x_*	Biomass concentration [g L^−1^]
HRT	Hydraulic Residence Time [d]
*P_A_*	Areal biomass productivity [g m^−2^ d^−1^]
*P_x_*	Volumetric biomass productivity [g L^−1^ d^−1^]
*Q*	volumetric flow rate [mL^−1^ d^−1^]
*r_Ai_*	Areal removal rate [mg L^−1^ m^2^ d^−1^]
*r_i_*	Removal rate [mg L^−1^ d^−1^]
*W*	Depth of reactor [m^2^]
%*C_i,bio_*	Fraction of compound internalized by the biomass but released after cell disruption [a.u.]
%*D_i_*	Fraction of compound not detectable in the sample [a.u]
%*R_i_*	Fraction of compound removed from the medium, but not found in the supernatant [a.u.]

## Abbreviations

CSTR	Continuously Stirred Tank Reactor
DO	Dissolved Oxygen
DW	Dry Weight
ESI	Electrospray Ionization
OPR	Oxygen Production Rate
OCR	Oxygen Consumption Rate
PFAS	Per- and poly-FluoroAlkyl Substances
PFOA	Perfluorooctanoic acid
PFOS	Perfluorooctane sulfonate

## References

[1] ITRC PFAS Fact Sheets PFAS. https://pfas-1.itrcweb.org/.

[2] Buck R.C., Franklin J., Berger U., Conder J.M., Cousins I.T., de Voogt P., Jensen A.A., Kannan K., Mabury S.A., van Leeuwen S.P. (2011). Perfluoroalkyl and Polyfluoroalkyl Substances in the Environment: Terminology, Classification, and Origins. Integr. Environ. Assess. Manag..

[3] Parsons J.R., Sáez M., Dolfing J., de Voogt P., Whitacre D.M. (2008). Biodegradation of Perfluorinated Compounds. Reviews of Environmental Contamination and Toxicology.

[4] Wang S., Yang Q., Chen F., Sun J., Luo K., Yao F., Wang X., Wang D., Li X., Zeng G. (2017). Photocatalytic Degradation of Perfluorooctanoic Acid and Perfluorooctane Sulfonate in Water: A Critical Review. Chem. Eng. J..

[5] Ahrens L., Bundschuh M. (2014). Fate and Effects of Poly- and Perfluoroalkyl Substances in the Aquatic Environment: A Review. Environ. Toxicol. Chem..

[6] Rayne S., Forest K. (2009). Perfluoroalkyl Sulfonic and Carboxylic Acids: A Critical Review of Physicochemical Properties, Levels and Patterns in Waters and Wastewaters, and Treatment Methods. J. Environ. Sci. Health Part A.

[7] World Health Organization (2017). Keeping Our Water Clean: The Case of Water Contamination in the Veneto Region, Italy.

[8] Pitter G., Da Re F., Canova C., Barbieri G., Zare Jeddi M., Daprà F., Manea F., Zolin R., Bettega A.M., Stopazzolo G. (2020). Serum Levels of Perfluoroalkyl Substances (PFAS) in Adolescents and Young Adults Exposed to Contaminated Drinking Water in the Veneto Region, Italy: A Cross-Sectional Study Based on a Health Surveillance Program. Environ. Health Perspect..

[9] Commission Regulation (EU) 2017/1510 Amending the Appendices to Annex XVII to Regulation (EC) No. 1907/2006 of the European Parliament and of the Council Concerning the Registration, Evaluation, Authorisation and Restriction of Chemicals (REACH) as Regards CMR Substances. https://www.ecolex.org/details/legislation/commission-regulation-eu-20171510-amending-the-appendices-to-annex-xvii-to-regulation-ec-no-19072006-of-the-european-parliament-and-of-the-council-concerning-the-registration-evaluation-authorisation-and-restriction-of-chemicals-reach-as-regards-cmr-substances-lex-faoc170309/.

[10] Mogensen U.B., Grandjean P., Nielsen F., Weihe P., Budtz-Jørgensen E. (2015). Breastfeeding as an Exposure Pathway for Perfluorinated Alkylates. Environ. Sci. Technol..

[11] Roth K., Imran Z., Liu W., Petriello M.C. (2020). Diet as an Exposure Source and Mediator of Per- and Polyfluoroalkyl Substance (PFAS) Toxicity. Front. Toxicol..

[12] Jackson T.W., Scheibly C.M., Polera M.E., Belcher S.M. (2021). Rapid Characterization of Human Serum Albumin Binding for Per- and Polyfluoroalkyl Substances Using Differential Scanning Fluorimetry. Environ. Sci. Technol..

[13] Di Nisio A., Foresta C. (2019). Water and Soil Pollution as Determinant of Water and Food Quality/Contamination and Its Impact on Male Fertility. Reprod. Biol. Endocrinol. RBE.

[14] Di Nisio A., Sabovic I., Valente U., Tescari S., Rocca M.S., Guidolin D., Dall’Acqua S., Acquasaliente L., Pozzi N., Plebani M. (2019). Endocrine Disruption of Androgenic Activity by Perfluoroalkyl Substances: Clinical and Experimental Evidence. J. Clin. Endocrinol. Metab..

[15] Per- and Polyfluoroalkyl Substance Toxicity and Human Health Review: Current State of Knowledge and Strategies for Informing Future Research-Fenton-2021-Environmental Toxicology and Chemistry-Wiley Online Library. https://setac.onlinelibrary.wiley.com/doi/10.1002/etc.4890.

[16] Zeng Z., Song B., Xiao R., Zeng G., Gong J., Chen M., Xu P., Zhang P., Shen M., Yi H. (2019). Assessing the Human Health Risks of Perfluorooctane Sulfonate by In Vivo and In Vitro Studies. Environ. Int..

[17] Li K., Gao P., Xiang P., Zhang X., Cui X., Ma L.Q. (2017). Molecular Mechanisms of PFOA-Induced Toxicity in Animals and Humans: Implications for Health Risks. Environ. Int..

[18] Kim K.Y., Ekpe O.D., Lee H.-J., Oh J.-E. (2020). Perfluoroalkyl Substances and Pharmaceuticals Removal in Full-Scale Drinking Water Treatment Plants. J. Hazard. Mater..

[19] Wanninayake D.M. (2021). Comparison of Currently Available PFAS Remediation Technologies in Water: A Review. J. Environ. Manage..

[20] Liu L., Bilal M., Duan X., Iqbal H.M.N. (2019). Mitigation of Environmental Pollution by Genetically Engineered Bacteria—Current Challenges and Future Perspectives. Sci. Total Environ..

[21] Moreira I.S., Amorim C.L., Murphy C.D., Castro P.M.L., Prasad R., Aranda E. (2018). Strategies for Biodegradation of Fluorinated Compounds. Approaches in Bioremediation: The New Era of Environmental Microbiology and Nanobiotechnology.

[22] Shahsavari E., Rouch D., Khudur L.S., Thomas D., Aburto-Medina A., Ball A.S. (2021). Challenges and Current Status of the Biological Treatment of PFAS-Contaminated Soils. Front. Bioeng. Biotechnol..

[23] Rylott E.L., Bruce N.C. (2020). How Synthetic Biology Can Help Bioremediation. Curr. Opin. Chem. Biol..

[24] Azubuike C.C., Chikere C.B., Okpokwasili G.C. (2016). Bioremediation Techniques–Classification Based on Site of Application: Principles, Advantages, Limitations and Prospects. World J. Microbiol. Biotechnol..

[25] Sayler G.S., Ripp S. (2000). Field Applications of Genetically Engineered Microorganisms for Bioremediation Processes. Curr. Opin. Biotechnol..

[26] Hagemann M., Hess W.R. (2018). Systems and Synthetic Biology for the Biotechnological Application of Cyanobacteria. Curr. Opin. Biotechnol..

[27] Wang J., Wang S. (2016). Removal of Pharmaceuticals and Personal Care Products (PPCPs) from Wastewater: A Review. J. Environ. Manag..

[28] Trentin G., Bertucco A., Sforza E. (2019). Mixotrophy in *Synechocystis* sp. for the Treatment of Wastewater with High Nutrient Content: Effect of CO_2_ and Light. Bioprocess Biosyst. Eng..

[29] Rippka R., Deruelles J., Waterbury J.B., Herdman M., Stanier R.Y. (1979). Generic Assignments, Strain Histories and Properties of Pure Cultures of Cyanobacteria. Microbiology.

[30] Sforza E., Pastore M., Barbera E., Bertucco A. (2019). Respirometry as a Tool to Quantify Kinetic Parameters of Microalgal Mixotrophic Growth. Bioprocess Biosyst. Eng..

[31] Nakayama S.F., Yoshikane M., Onoda Y., Nishihama Y., Iwai-Shimada M., Takagi M., Kobayashi Y., Isobe T. (2019). Worldwide Trends in Tracing Poly- and Perfluoroalkyl Substances (PFAS) in the Environment. TrAC Trends Anal. Chem..

[32] Fabris M., Abbriano R.M., Pernice M., Sutherland D.L., Commault A.S., Hall C.C., Labeeuw L., McCauley J.I., Kuzhiuparambil U., Ray P. (2020). Emerging Technologies in Algal Biotechnology: Toward the Establishment of a Sustainable, Algae-Based Bioeconomy. Front. Plant Sci..

[33] Tran K.M., Lee H.-M., Thai T.D., Shen J., Eyun S., Na D. (2021). Synthetically Engineered Microbial Scavengers for Enhanced Bioremediation. J. Hazard. Mater..

[34] Rossi S., Sforza E., Pastore M. (2020). Photo-Respirometry to Shed Light on Microalgae-Bacteria Consortia—A Review. Rev. Environ. Sci. Biotechnol..

[35] Phong Vo H.N., Ngo H.H., Guo W., Hong Nguyen T.M., Li J., Liang H., Deng L., Chen Z., Hang Nguyen T.A. (2020). Poly-and Perfluoroalkyl Substances in Water and Wastewater: A Comprehensive Review from Sources to Remediation. J. Water Process. Eng..

[36] Huang F., Hedman E., Funk C., Kieselbach T., Schröder W.P., Norling B. (2004). Isolation of Outer Membrane of *Synechocystis* sp. PCC 6803 and Its Proteomic Characterization. Mol. Cell. Proteom..

[37] Commissione Parlamentare Di Inchiesta Sulle Attività Illecite Connesse al Ciclo Di Rifiuti e Su Illeciti Ambientali Ad Essi Correlati. https://www.senato.it/service/PDF/PDFServer/BGT/1064090.pdf.

[38] Sforza E., Barbera E., Bertucco A. (2015). Improving the Photoconversion Efficiency: An Integrated Photovoltaic-Photobioreactor System for Microalgal Cultivation. Algal Res..

[39] Sforza E., Calvaruso C., Meneghesso A., Morosinotto T., Bertucco A. (2015). Effect of Specific Light Supply Rate on Photosynthetic Efficiency of Nannochloropsis Salina in a Continuous Flat Plate Photobioreactor. Appl. Microbiol. Biotechnol..

[40] Liu W., Chen S., Quan X., Jin Y.-H. (2008). Toxic Effect of Serial Perfluorosulfonic and Perfluorocarboxylic Acids on the Membrane System of a Freshwater Alga Measured by Flow Cytometry. Environ. Toxicol. Chem..

[41] Fitzgerald N.J.M., Wargenau A., Sorenson C., Pedersen J., Tufenkji N., Novak P.J., Simcik M.F. (2018). Partitioning and Accumulation of Perfluoroalkyl Substances in Model Lipid Bilayers and Bacteria. Environ. Sci. Technol..

[42] Boudreau T.M., Sibley P.K., Mabury S.A., Muir D.G.C., Solomon K.R. (2003). Laboratory Evaluation of the Toxicity of Perfluorooctane Sulfonate (PFOS) on Selenastrum Capricornutum, Chlorella Vulgaris, Lemna Gibba, Daphnia Magna, and Daphnia Pulicaria. Arch. Environ. Contam. Toxicol..

[43] Hu C., Luo Q., Huang Q. (2014). Ecotoxicological Effects of Perfluorooctanoic Acid on Freshwater Microalgae Chlamydomonas Reinhardtii and Scenedesmus Obliquus. Environ. Toxicol. Chem..

[44] Rosal R., Rodea-Palomares I., Boltes K., Fernández-Piñas F., Leganés F., Petre A. (2010). Ecotoxicological Assessment of Surfactants in the Aquatic Environment: Combined Toxicity of Docusate Sodium with Chlorinated Pollutants. Chemosphere.

[45] Niu Z., Na J., Xu W., Wu N., Zhang Y. (2019). The Effect of Environmentally Relevant Emerging Per- and Polyfluoroalkyl Substances on the Growth and Antioxidant Response in Marine *Chlorella* sp.. Environ. Pollut..

[46] Li Y., Liu X., Zheng X., Yang M., Gao X., Huang J., Zhang L., Fan Z. (2021). Toxic Effects and Mechanisms of PFOA and Its Substitute GenX on the Photosynthesis of Chlorella Pyrenoidosa. Sci. Total Environ..

[47] USA EPA Reducing PFAS in Drinking Water with Treatment Technologies. https://www.epa.gov/sciencematters/reducing-pfas-drinking-water-treatment-technologies.

[48] El Gamal M., Mousa H.A., El-Naas M.H., Zacharia R., Judd S. (2018). Bio-Regeneration of Activated Carbon: A Comprehensive Review. Sep. Purif. Technol..

[49] Gobelius L., Lewis J., Ahrens L. (2017). Plant Uptake of Per- and Polyfluoroalkyl Substances at a Contaminated Fire Training Facility to Evaluate the Phytoremediation Potential of Various Plant Species. Environ. Sci. Technol..

[50] Butzen M.L., Wilkinson J.T., McGuinness S.R., Amezquita S., Peaslee G.F., Fein J.B. (2020). Sorption and Desorption Behavior of PFOS and PFOA onto a Gram-Positive and a Gram-Negative Bacterial Species Measured Using Particle-Induced Gamma-Ray Emission (PIGE) Spectroscopy. Chem. Geol..

[51] Zhao W., Zitzow J.D., Ehresman D.J., Chang S.-C., Butenhoff J.L., Forster J., Hagenbuch B. (2015). Na+/Taurocholate Cotransporting Polypeptide and Apical Sodium-Dependent Bile Acid Transporter Are Involved in the Disposition of Perfluoroalkyl Sulfonates in Humans and Rats. Toxicol. Sci..

[52] Mudumbi J.B.N., Ntwampe S.K.O., Mekuto L., Itoba-Tombo E.F., Matsha T.E. (2017). Are Aquaporins (AQPs) the Gateway That Conduits Nutrients, Persistent Organic Pollutants and Perfluoroalkyl Substances (PFASs) into Plants?. Springer Sci. Rev..

[53] Colosi L.M., Pinto R.A., Huang Q., Weber W.J. (2009). Peroxidase-Mediated Degradation of Perfluorooctanoic Acid. Environ. Toxicol. Chem..

[54] Luo Q., Liang S., Huang Q. (2018). Laccase Induced Degradation of Perfluorooctanoic Acid in a Soil Slurry. J. Hazard. Mater..

[55] Li Y., Yue Y., Zhang H., Yang Z., Wang H., Tian S., Wang J., Zhang Q., Wang W. (2019). Harnessing Fluoroacetate Dehalogenase for Defluorination of Fluorocarboxylic Acids: In Silico and In Vitro Approach. Environ. Int..

[56] Huang S., Jaffé P.R. (2019). Defluorination of Perfluorooctanoic Acid (PFOA) and Perfluorooctane Sulfonate (PFOS) by *Acidimicrobium* sp. Strain A6. Environ. Sci. Technol..

[57] Luo Q., Lu J., Zhang H., Wang Z., Feng M., Chiang S.-Y.D., Woodward D., Huang Q. (2015). Laccase-Catalyzed Degradation of Perfluorooctanoic Acid. Environ. Sci. Technol. Lett..

[58] Morsi R., Bilal M., Iqbal H.M.N., Ashraf S.S. (2020). Laccases and Peroxidases: The Smart, Greener and Futuristic Biocatalytic Tools to Mitigate Recalcitrant Emerging Pollutants. Sci. Total Environ..

