# Supplementary material for: Bioremediation of Per- and Poly-Fluoroalkyl Substances (PFAS) by Synechocystis sp. PCC 6803: A Chassis for a Synthetic Biology Approach
Source: Life (Basel). 2021 Nov 26;11(12):1300. doi: 10.3390/life11121300 (PMC8707875; doi:10.3390/life11121300)
Supplement: Supplementary file 1 [file life-11-01300-s001.zip › life-1469295-supplementary.pdf]

life

MDPI

Article

Bioremediation of Per- and Poly-Fluoroalkyl Substances (PFAS) by *Synechocystis* sp. PCC 6803: A Chassis for a Synthetic Biology Approach

Francesca Marchetto [1,†], Marco Roverso [2,†], Davide Righetti [1,3], Sara Bogialli [2], Francesco Filippini [3], Elisabetta Bergantino [3] and Eleonora Sforza [1,*]

1 Department of Industrial Engineering DII, University of Padova, 35131 Padova, Italy; marchetto.francesca95@gmail.com (F.M.); davide.righetti.1@studenti.unipd.it (D.R.)
2 Department of Chemical Sciences, University of Padova, 35131 Padova, Italy; marco.roverso@unipd.it (M.R.); sara.bogialli@unipd.it (S.B.)
3 Department of Biology, University of Padova, 35131 Padova, Italy; francesco.filippini@unipd.it (F.F.); elisabetta.bergantino@unipd.it (E.B.)
* Correspondence: eleonora.sforza@unipd.it; Tel.: +39-049-827-5267
† These authors contributed equally to this work.

Supplementary Material:

Table S1. Candidate *Synechocystis* proteins possibly involved in PFASs transport and/or metabolism. * Found in *Synechocystis* PCC 6803.

Name	Biochemical function	Sequence(s)	Accession	% similarity
Na+-taurocholate cotransporting polypeptide (NTCP)	Bile acid:sodium symporter	8	WP_028947894.1<br>WP_010872629.1<br>WP_162328214.1<br>MBE9176556.1<br>WP_194071296.1<br>WP_190599003.1<br>WP_194019469.1<br>WP_194014785.1	27-29% to Human NTCP, NTCP2, NTCP4 and NTCP7
ATP binding cassette transporter C family member (ABCC)	ATP-binding cassette domain-containing protein	40	WP_194072738.1<br>WP_194018933.1<br>WP_190596069.1<br>WP_194015207.1<br>WP_162327634.1<br>WP_199303459.1<br>WP_190597748.1<br>WP_162327599.1<br>WP_194018815.1<br>WP_194013374.1<br>WP_194013374.1<br>WP_162327849.1<br>WP_190598847.1<br>WP_194014885.1<br>WP_194018910.1<br>WP_190597363.1<br>WP_194073247.1<br>WP_162329505.1<br>WP_162327719.1	23–42% to *Arabidopsis thaliana* ABCC2

			WP_194015044.1<br>WP_162327093.1<br>WP_194073659.1<br>WP_194016394.1<br>WP_028946876.1<br>WP_194015365.1<br>WP_194017886.1<br>WP_194017886.1<br>WP_162328301.1<br>WP_194015754.1<br>WP_194020455.1<br>WP_190596393.1<br>WP_194072749.1<br>WP_190599643.1<br>WP_162329061.1<br>WP_190597943.1<br>WP_162327446.1<br>WP_194015181.1<br>WP_190596040.1<br>MBE9175611.1<br>WP_194020078.1	
	ABC transporter ATP-binding protein	44	MBE9175251.1<br>WP_028948219.1<br>WP_010871936.1<br>MBE9174824.1<br>WP_028948200.1<br>WP_010871895.1<br>WP_009633118.1<br>WP_028947992.1<br>WP_028947381.1<br>WP_071822647.1<br>WP_009630252.1<br>WP_009632343.1<br>WP_010873840.1<br>WP_009632727.1<br>WP_028954270.1<br>WP_051738793.1<br>WP_009632913.1<br>WP_028949030.1<br>WP_010871235.1<br>WP_010872070.1<br>WP_028947037.1<br>WP_009632475.1<br>WP_028949033.1<br>WP_009631388.1<br>WP_009632594.1<br>WP_010871241.1<br>WP_162329293.1<br>WP_028947003.1<br>WP_010874137.1<br>WP_194020635.1	23–36% to *Arabidopsis thaliana* ABCC2

			MBE9174726.1<br>WP_010872849.1<br>WP_051738954.1<br>WP_051482607.1<br>WP_009633356.1<br>WP_028949247.1<br>AIE73207.1<br>WP_009631439.1<br>MBE9174324.1<br>WP_028954202.1<br>WP_009633629.1<br>WP_028947788.1<br>WP_010873804.1<br>WP_009633685.1	
	peptidase domain-containing ABC transporter	5	MBE9175630.1<br>WP_010872887.1<br>WP_028948277.1<br>WP_009630782.1<br>WP_010874249.1	23–27% to *Arabidopsis thaliana* ABCC2
	type I secretion system permease/ATPase	2	WP_194013658.1<br>WP_162328330.1	25–28% to *Arabidopsis thaliana* ABCC2
	ABC transporter *	3	AGF51262.1<br>QHV01854.1<br>ALJ68769.1*<br>AGF51262.1*<br>ALJ69526.1*<br>WP_014407101.1*	27–29% to *Arabidopsis thaliana* ABCC2
	TOBE-like domain-containing protein	2	WP_190598704.1<br>WP_009631742.1	30–34% to *Arabidopsis thaliana* ABCC2
	Multidrug resistance ABC transporter ATP-binding and permease protein	1	AIE74659.1	36% to *Arabidopsis thaliana* ABCC2
	cyclic nucleotide-regulated ABC bacteriocin/lantibiotic exporter protein	1	AVH79543.1	26% to *Arabidopsis thaliana* ABCC2
	ATP-binding protein of ABC transporter	1	AIE73432.1	31% to *Arabidopsis thaliana* ABCC2
Aquaporin (aqp)	Aquaporin	8	WP_162328075.1<br>WP_028946756.1<br>MBE9176180.1<br>WP_194015914.1<br>WP_190599016.1<br>WP_009631497.1<br>WP_194073056.1<br>WP_009633089.1	37–100% to *Synechocystis* PCC 6803 aqpZ
Laccase	Multicopper oxidase domain-containing protein	1	WP_194014464.1	24% to *Pleurotus ostreatus* laccase POX1
Fluoroacetate dehalogenase	Alpha/beta hydrolase	19	WP_009633998.1<br>WP_009634094.1<br>WP_028949175.1<br>WP_010872272.1	25–39% to *Burkholderia sp.* Fac-dex

			WP_028946933.1<br>WP_028954141.1<br>WP_010871769.1<br>WP_106907929.1<br>WP_009634549.1<br>WP_009631931.1<br>WP_038531141.1<br>AIE75980.1<br>WP_010872160.1<br>QHV01713.1<br>WP_009630128.1<br>WP_010873707.1<br>WP_028948001.1<br>WP_038021267.1<br>QHV01001.1	
	Alpha/beta fold hydrolase	53	WP_194019009.1<br>WP_194072249.1<br>WP_190599725.1<br>WP_162327898.1<br>WP_194013416.1<br>WP_190598114.1<br>WP_194020943.1<br>WP_194017332.1<br>WP_162327504.1<br>WP_038020775.1<br>WP_028947035.1<br>MBE9175246.1<br>WP_009631087.1<br>WP_190598236.1<br>WP_194019539.1<br>WP_162327488.1<br>WP_194073349.1<br>WP_162327505.1<br>WP_009630182.1<br>WP_190598115.1<br>WP_010871750.1<br>WP_010871770.1<br>WP_194020945.1<br>WP_194017334.1<br>WP_194015193.1<br>WP_028947752.1<br>MBE9176742.1<br>WP_194020915.1<br>MBD2655166.1<br>WP_190599431.1<br>WP_194072888.1<br>WP_009630376.1<br>WP_162327789.1<br>WP_194018987.1<br>WP_194014523.1<br>WP_199303425.1	22–40% to *Burkholderia sp.* Fac-dex

	WP_190599423.1
	MBE9176468.1
	WP_194072577.1
	WP_010872152.1
	WP_199303557.1
	WP_194018983.1
	WP_190599278.1
	WP_194013777.1
	WP_194019704.1
	MBE9175978.1
	WP_028946321.1
	WP_199309777.1
	WP_194014536.1
	WP_162327783.1
	WP_080504060.1
	WP_162329611.1
	WP_038530520.1
